# Iron Carbide@Carbon Nanocomposites: A Tool Box of Functional Materials

**DOI:** 10.3390/ma12020323

**Published:** 2019-01-21

**Authors:** Chiara Defilippi, Mariam Omar Ali Mukadam, Sabina Alexandra Nicolae, Martin Richard Lees, Cristina Giordano

**Affiliations:** 1 School of Biological and Chemical Sciences, Chemistry Department, Queen Mary University of London, Mile End Road, London E1 4NS, UK; c.defilippi@qmul.ac.uk (C.D.); m.mukadam@se14.qmul.ac.uk (M.O.A.M.); 2 School of Engineering and Materials Science, Queen Mary University of London, Mile End Road, London E1 4NS, UK; sabina.nicolae@qmul.ac.uk; 3 Department of Physics, University of Warwick, Coventry CV4 7AL, UK; m.r.lees@warwick.ac.uk

**Keywords:** metallic ceramics, nanocomposites, urea glass route, iron carbide, chitosan

## Abstract

Iron carbide (Fe_3_C) is a ceramic magnetic material with high potential for applications in different fields, including catalysis, medicine imaging, coatings, and sensors. Despite its interesting properties, it is still somehow largely unexplored, probably due to challenging synthetic conditions. In this contribution, we present a sol-gel-based method that allows preparing different Fe_3_C@C nanocomposites with tailored properties for specific applications, in particular, we have focused on and discussed potential uses for adsorption of noxious gas and waste removal. Nanocomposites were prepared using readily available and “green” sources, such as urea, simple and complex sugars, and chitosan. The nanocomposite prepared from chitosan was found to be more efficient for CO_2_ uptake, while the sample synthetized from cellulose had optimal capability for dye absorption and waste oil removal from water.

## 1. Introduction

The powerful combination of iron and carbon to improve material performances has been known since antiquity. According to a legend, the art of the carburization of iron might have been developed by an Anatolian population called Chalybes, who could have also forged the famous sword of Julius Cesar. Mythology aside, it was discovered that metallurgy employing combinations of iron and carbon dates back even to prehistory when meteorites were used as the first sources of iron [1]. Beads found in tomb 67 at Gerzeh, which were made by using meteoritic iron as the metal source, represent one of the earliest examples of exploitation of iron in Egypt [2]. Even the superior hardness of Damascus sabre seems to arise from iron carbide nanoparticles reinforcing iron metal [3].

Nowadays, the best and most known representative of the iron/carbon winning combination is surely steel but, with the advent of nanoscience, the iron/carbon blend can be finely adjusted toward designed applications. In this way, besides a mere control of the elements ratio (as for the case of steel), we can prepare iron-based (nano)composites, as this study will also show, where carbon acts as functional matrix (contributing to a higher surface area, improved electron transport, and additional conductivity to the final system), while the iron-based phases represent the (nano)fillers, providing the final material with magnetic properties. Here, a whole tool box of possibility unfolds, where final composition, size, and morphology can be tailored for specific applications. The range of applications can be even broadened if, alongside pure iron, iron carbide is also considered. Iron carbide has high thermal and chemical resistance, and might also be used for applications where harsh conditions are required and where, usually, pure iron or iron oxides do not survive. Furthermore, in its most simple cementite form, it is less toxic than pure iron but, at the same time, more magnetic than iron oxide, and can, for instance, also be considered in nanomedicine, for example, as contrast agent for MRI and drug delivery [4]. For this reason, toxicity/biocompatibility was studied both in vitro and in vivo with cytotoxicity essays, involving several cells, such as macrophage, mouse fibroblast, human breast adenocarcinoma, and human ovarian carcinoma. These tests were performed on different iron carbides, such as Fe_7_C_3_, Fe_5_C_2_, and Fe_3_C, and did not show significant negative side effects [4,5]. Their magnetic properties make iron-based materials also easily recoverable after use, simply by applying an external magnetic field, thus favouring their reuse and waste collection [6].

Besides biomedical applications, iron carbide can be used as sensors, coatings [7], and catalysts in processes such as Fischer–Tropsch, ammonia decomposition [8,9], and CO_2_ hydrogenation [10].

Considering the crucial problems of environmental pollution from air and wastewater [11,12], chemical sensing and pollutant absorbent are surely important for the detection of noxious gases, such as CO_2_ [11]. Iron-based materials, such as iron oxides [12,13,14] are used as magnetic gas sensors in different applications [15].

Suitable functionalization of iron carbide nanoparticles can also be used for the preparation of novel magnetic fluids.

**Scheme 1:**
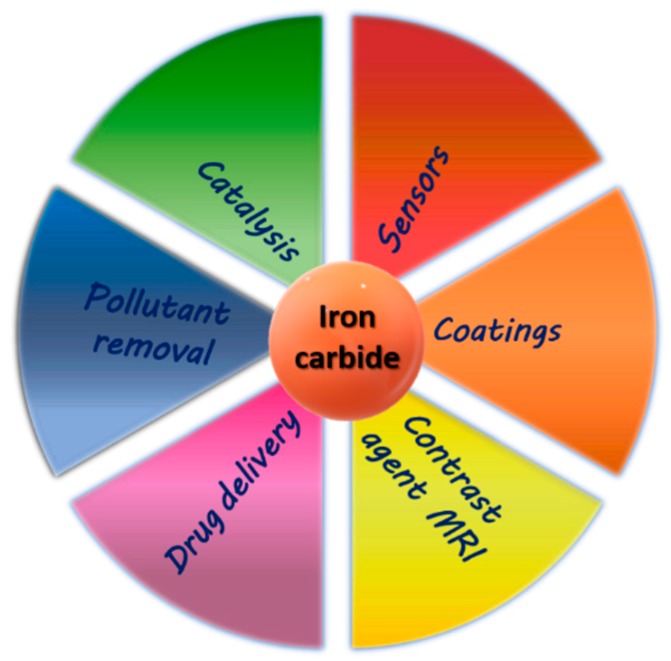
Overview of the manifold application of iron carbide-based materials.

An overall representation of iron carbide applications is reported in Scheme 1.

Despite its high potential, iron carbide-based materials are still not as broadly considered as the corresponding oxides or parental metal [16]. This is probably due to the difficulties related to their synthesis, such as chemical vapour deposition (CVD)-assisted pyrolysis [7], and ammonolysis, which require high temperatures, rendering iron carbide production more expensive but also hindering control over the size and morphology of the final product when prepared at the nanoscale [17,18].

In this paper, we present how iron carbide@carbon nanocomposites can be prepared and shaped to change the final material functionality, adjusting its composition (e.g., incorporating a useful amount of iron), size, and crystallinity, but also changing properties such as magnetism, surface area, absorption, and more, which in turn result in a material with higher potential for biomedical applications (e.g., in hyperthermia treatment), diagnostic (e.g., as a contrast agent in MRI), absorption of toxic molecules, sensor technology, catalytic efficiency, etc. Here, we will only show a few model examples, but the application range can be much broader. In particular, we have focused our attention in preparing materials with higher magnetization and/or larger surface area for future potential application in the detection of noxious gases, such as CO_2_, or absorption of pollutants such as inorganic dyes or scrap oils from water, keeping in mind that an effective gas sensor/adsorbent should be cost-effective and highly sensitive, to detect low concentrations. Here, nanocomposites with higher surface area promote contact surfaces to the analytes, and have increased sensitivity and gas adsorption compared to bulk samples [11,19].

To overcome the synthetic difficulties, we have employed a sustainable route, based on a sol-gel process, to prepare the nanocomposite, that allows using lower reaction temperatures compared to classical synthesis, and to adjust morphology, particle dimension, and surface area of the final material simply by varying the carbon source and reaction conditions [18]. 

## 2. Materials and Methods

### 2.1. Iron Carbide Synthesis

Iron(III) acetylacetonate (1 g, Fe(acac)_3_, Sigma-Aldrich 97%, F300, Gillingham, UK) was used as iron source and mixed with approximately 2.5 mL of ethanol to obtain a clear solution. The solution was then mixed with a suitable carbon source to obtain the final iron carbide upon heat treatment. As carbon source, several compounds were explored, going from small molecules such as urea (CO(NH_2_)_2_, Sigma-Aldrich 99%, U5128), to sugars such as D-glucose (analytical reagent grade, Fisher Scientific,10391201, Loughborough, UK), D(+)-sucrose (analytical reagent grade, Fisher Scientific, 10647421), and chitosan (*M*_W_: 100,000–300,000, VWR, ACRO349050500) but also a polymer-based sugar such as cellulose (Sigmacell, Type 20, 20 μm Sigma-Aldrich, S3504). The cellulose was heat treated prior to its utilization (21 °C/min ramping until 800 °C under N_2_ flow, no dwelling) to remove possibly absorbed molecules on its surface.

**Figure 1:**
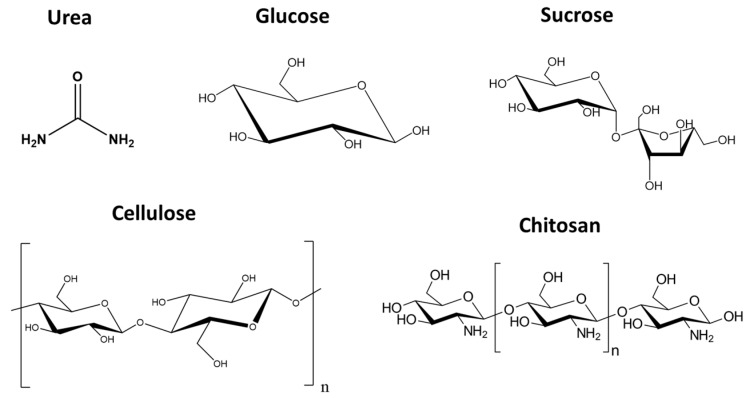
Molecular structures of the used carbon sources.

The carbon source strongly affects the texture of the carbon matrix, the composition of the final material but also its surface area and porosity. Specific synthetic details are reported in Table 1, while Figure 1 reports the chemical structure of the several carbon sources used.

For chitosan and cellulose samples, the percentage reported refers to wt % between iron precursors and carbon source.

**Scheme 2:**
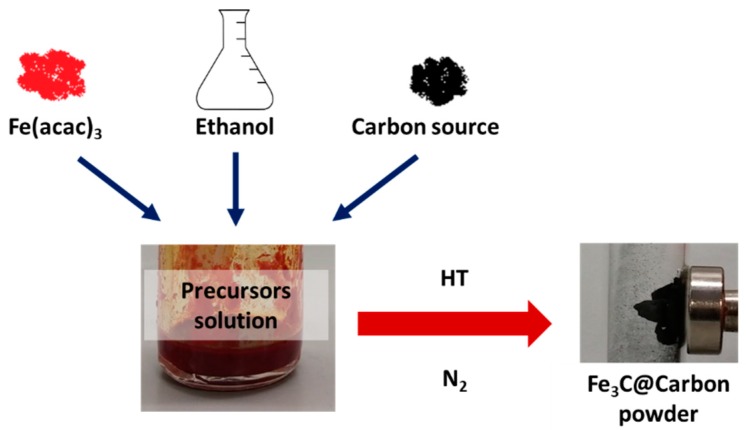
Overview of the synthesis of Fe_3_C samples.

The heat treatment (up to 800 °C for 2 h under nitrogen flow) leads to a black (matt or shiny depending on the carbon source) powder made of crystalline Fe_3_C and carbon, as ascertained by X-ray investigation. In some cases, the presence of Fe^0^ was also observed (see PXRD patterns vide infra). A representation of the procedure is reported in Scheme 2.

### 2.2. Dyes and Oil Uptake Procedure

Methyl orange (Sigma-Aldrich, ACS reagent, dye content 85%, 114,510) and methylene blue (Sigma-Aldrich, ≥97.0%, 66,720) solution were prepared by dissolving the powder of the dye in water to achieve the desired final concentration. Solutions were kept in the dark to reduce natural photodegradation.

As prepared samples powders (around 5 mg) were mixed with the dye solutions (5 mL) and UV–Vis measurements were performed over time to measure the dye concentration.

Vegetable oil (Tesco®, pure sunflower oil) was added to water containing the Fe_3_C-based sample and the affinity of the compound was recorded by visual inspection.

### 2.3. Techniques

To evaluate the crystallinity of the samples and the phases present, X-ray powder diffraction (PXRD) measurements were performed on XPERT Panalytical Empyrean Diffractometer (Cu Kα radiation, λ = 0.154 nm, at 45 kV and 40 mA, Malvern Panalytical Ltd, Royston, UK) equipped with an X’Celerator detector.

To obtain information about the morphology and large-scale homogeneity of the samples, scanning electron microscopy (SEM, Thermo Fisher Scientific, Hillsboro, USA) was performed on a FEI inspect F instrument. The samples were loaded on carbon-coated stubs and coated by sputtering an Au prior to imaging.

To observe the size and shape of the particles, transmission electron microscopy (TEM, JEOL (U.K.) Limited, London, UK) was performed on a JEOL 2010 operated at an acceleration voltage of 200 kV. Samples were ground and then suspended in ethanol. One drop of this suspension was put on a holey carbon-coated copper grid of 300 mesh and left to dry.

Elemental analysis was conducted using a Thermo Scientific FLASH 2000 Elemental Analyzer (Thermo Fisher Scientific, Hillsboro, USA). Information about the surface area/CO_2_ adsorption of the material were obtained using Quantachrome Nova 4200e (Anton Paar GmbH, Graz, Austria). N_2_ adsorption-desorption isotherms were measured at −196 °C. The samples were degassed at 150 °C under vacuum overnight, prior to the adsorption. BET theory was used to calculate surface area. For the CO_2_ adsorption experiments, the samples were degassed at 150 °C under vacuum overnight. Measurements were performed at 1 bar and 0 °C. 

Magnetic (M vs. H) measurements were performed using a Quantum Design Magnetic Property Measurement System (MPMS, Quantum Design, Inc., San Diego, USA) squid magnetometer. A small quantity of each sample was placed in a gel capsule and this capsule was then placed in a plastic drinking straw. M vs. H data were collected sweeping the magnetic field from +50 kOe (5 T) to −50 kOe, and then back to +50 kOe at *T* = 300 K. Ultraviolet-visible (UV–Vis, Agilent Technologies LDA UK Limited, Stockport, UK) spectroscopy measurements were performed using Cary 100 Agilent spectrophotometer to measure the absorbance of the dye solutions.

## 3. Results and Discussion

**Figure 2:**
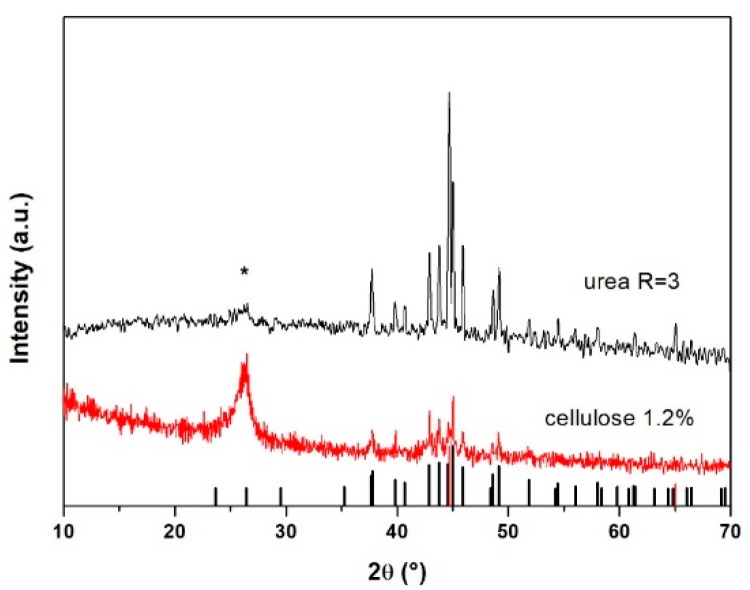
PXRD patterns of samples prepared using urea (*R* = 3, upper pattern) and cellulose (1.2% downer pattern). The expected pattern from the database for pure Fe_3_C and Fe^0^ are reported as vertical lines for comparison (Fe_3_C, black lines, ICDD: 03-065-2413 and Fe^0^ red lines, ICDD: 01-087-0721. Marked peak (*) is attributed to carbon (ICDD 01-077-7164).

The crystal structure of the final material was ascertained by PXRD study, using ICDD (PDF4+ version) database. Using urea as carbon source, crystalline iron carbide was obtained (Figure 2, upper pattern) alongside a small amount of amorphous carbon (* marked peak) and Fe^0^. On the other hand, when cellulose was used as C source, the peak of carbon is more defined and prominent, indicating the formation of a quasi-crystalline (possibly turbostratic) phase, alongside the pattern of iron carbide. In this case, the presence of metallic iron was not observed. For the cellulose sample, the increased background at lower angles could be due to the presence of amorphous carbon.

**Figure 3:**
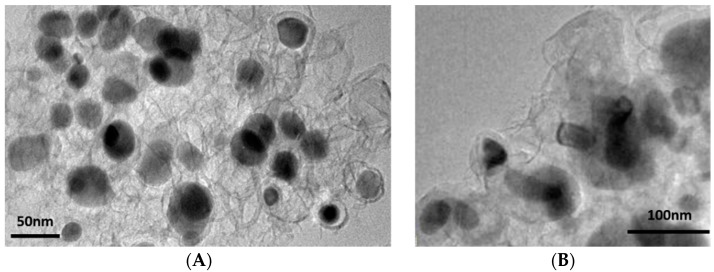
TEM images of samples prepared with (**A**) urea *R* = 3 and (**B**) cellulose. Particle dimension histograms are reported in Figure S1.

The presence of the carbon matrix seems to affect, also, the final size of the iron carbide nanoparticles, which appeared to be bigger than in the presence of urea (as ascertained by Scherrer calculation of the mean crystallite size and also observed by TEM investigation, Figure 3). The presence of the carbon matrix drastically increases the surface area of the sample prepared with cellulose, which was determined to be above 200 m^2^/g, against the lesser value of 50 m^2^/g for the sample prepared using urea (see Table 2). On the other hand, the magnetization of the sample prepared via urea was much higher than that observed for the cellulose-derived sample (100 emu/g vs. <20 emu/g, see Table S1), which means a better suitability of the former for application where magnetization plays the main role, while cellulose-derived samples are more suitable for applications where surface area is crucial.

In the case of urea and cellulose, from the TEM images, it can be seen that the inorganic nanoparticles (metallic in nature, i.e., with higher electron density and therefore showing higher contrast) are distributed on a matrix, possibly turbostratic carbon, in line with PXRD observation (broad carbon peak around 26° 2θ). These results are similar to what reported previously, where the catalytic graphitization of carbon by iron was also observed [20].

Regarding the SEM study, samples prepared using urea and cellulose as carbon source form a quite compact structure, where “wrapped” structures can be also observed (Figure S2). These rods could be due to thin sheets of iron/carbon, which wrap into rods, as previously observed [9]. 

In order to investigate the role of the carbon source in the formation of a carbon functional matrix, cellulose was replaced with simple sugar molecules, such as glucose and sucrose.

**Figure 4:**
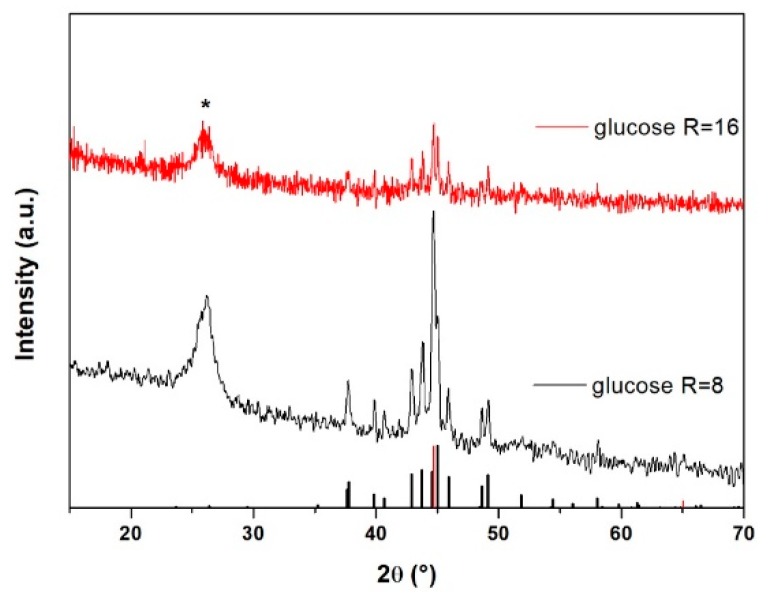
PXRD pattern of samples prepared using glucose as carbon source with two different glucose/Fe molar ratios (*R*). The expected pattern from the database for pure Fe_3_C and Fe^0^ are also reported as vertical lines for comparison (Fe_3_C, black lines, ICDD: 03-065-2413 and Fe^0^ red lines, ICDD: 01-087-0721. The marked peak (*) is attributed to carbon (ICDD 01-077-7164).

A similar trend to the cellulose-derived samples was observed when glucose was used as carbon source. Also in this case, both iron carbide and carbon peaks are observed (Figure 4). From Figure 4, it can be seen that for higher *R*, the carbon peak is less intense and broader while, for lower urea/metal ratio (*R*), residual Fe^0^ is observed.

Although glucose is a reducing sugar, a higher amount (i.e., higher *R*) hinders Fe^0^ formation, possibly towards the formation of ‘reduced’ phases.

The presence of the carbon matrix imparts, to the glucose-derived samples, a higher surface area (>200 m^2^/g, see Table 2). 

**Figure 5:**
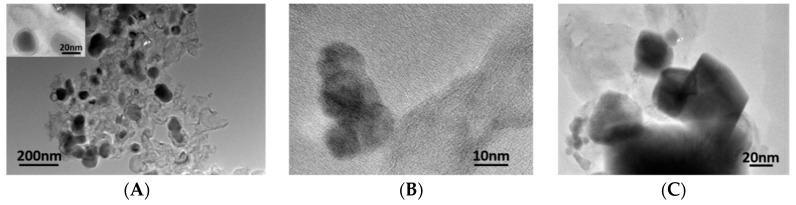
TEM images of samples prepared using glucose as carbon source with different glucose/iron molar ratio (**A**) and (**B**) *R* = 8 and (**C**) *R* = 16. Particle dimension histograms are reported in Figure S1.

TEM investigation revealed, for both ratios (*R* = 8 and 16), the formation of medium-sized nanoparticles (20–60 nm on average), mostly agglomerated and ill-shaped, dispersed in the carbon matrix. As expected from the PXRD pattern (see Figure 4), for *R* = 16, nanoparticles are slightly smaller than for *R* = 8, but the presence of bigger and ill-shaped aggregates can be also seen (see Figure 5). Interestingly, the lower degree of crystallization for the carbon phase in *R* = 16 seems to also affect the nanoparticles’ morphology, possibly due to a lack of ‘protection’. In fact, for *R* = 8, a graphitic carbon shell around the particles was observed, which might act as a protective coating (see inset Figure 5A).

SEM images of the glucose-derived samples are reported in Figure S3, where a rod-like structure can be observed (similar to those observed for the case of the urea-derived sample). Those “needles” are absent in the sample prepared with higher urea/metal ratio (*R* = 16). 

**Figure 6:**
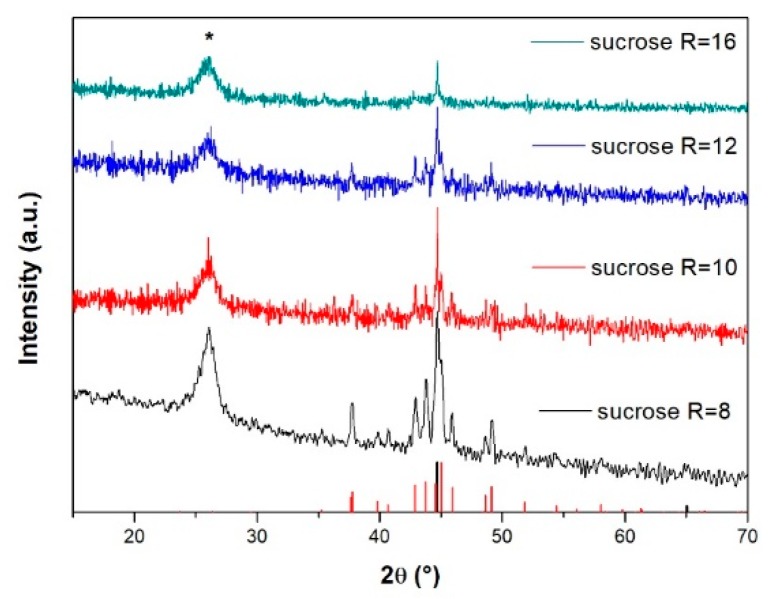
PXRD pattern of samples prepared with sucrose at different molar ratios (*R*). The expected pattern from the database for pure Fe_3_C and Fe^0^ are also reported as vertical lines for comparison (Fe_3_C, red lines, ICDD: 03-065-2413, and Fe^0^ black lines, ICDD: 01-087-0721. The marked peak (*) is attributed to carbon (ICDD 01-077-7164).

Interestingly, a completely different trend is observed when replacing glucose with sucrose. The use of sucrose as carbon source allows for the formation of iron carbide only at a lower sugar/metal molar ratio, although carbon and a significant amount of iron zero were formed, as ascertained in all cases by PXRD investigation (see Figure 6). The residual metal iron increases with higher *R* (i.e., the initial sugar amount) to the detriment of the carbide phase, which disappears completely for *R* ≥ 16. Alongside with increasing *R*, the peak of carbon also reduces in intensity and becomes broader, indicating that the formation of the iron phase has not supported the graphitization of carbon. The reason behind this trend requires a dedicated study and specific equipment (e.g., EXAFS for in situ measurements), and this is in progress. In any case, SEM study reveals the homogeneity of the samples (see Figure S4).

**Figure 7:**
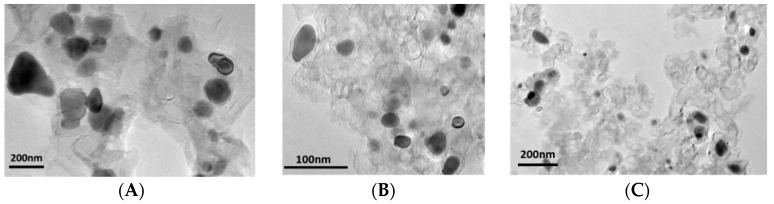
TEM images of samples prepared with sucrose (**A**) *R* = 8, (**B**) *R* = 10, and (**C**) *R* = 12. Particle dimension histograms are reported in Figure S1.

From TEM analysis (Figure 7) the particles of sample *R* = 8 (Figure 7A) are mostly agglomerated, while, by increasing R, the particles become smaller and less aggregated, for all ratios, and the particles are surrounded by the carbon matrix.

In order to understand whether the initial complexation of the iron source may play a role, chitosan was also explored as a carbon source. Thanks to the presence of NH_2_ groups, a stronger bonding between chitosan and Fe^3+^ was expected [21]. Furthermore, chitosan decomposes at lower temperatures compared to cellulose [22].

**Figure 8:**
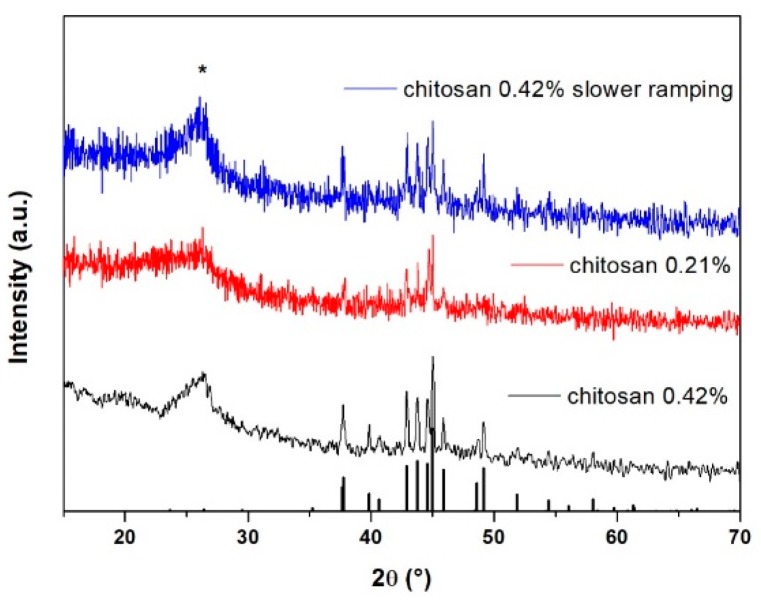
PXRD pattern of samples prepared with chitosan. The expected pattern from the database for pure Fe_3_C is also reported as vertical lines for comparison (Fe_3_C, black lines, ICDD: 03-065-2413). Marked peak (*) is attributed to carbon (ICDD 01-077-7164).

As expected, all samples prepared with chitosan form pure Fe_3_C, and no Fe^0^ was observed, although the chitosan/iron molar ratio had no influence on the final composition of the sample, as well as the heating rate. PXRD patterns of the chitosan samples are reported in Figure 8.

**Figure 9:**
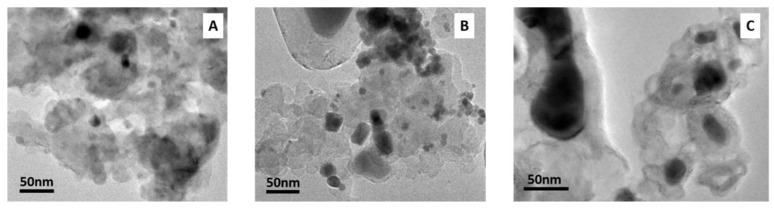
TEM images of samples prepared with different amounts of chitosan (**A**) 0.42%, (**B**) 0.21%, and (**C**) 0.42%, slower heat ramping. Particle dimension histograms are reported in Figure S1.

On the other hand, the sample’s lack of homogeneity and carbon phase looks less defined, compared to the cases described previously. Fe_3_C nanoparticles (darker spot in the TEM images) are smaller and spheroidal (Figure 9).

SEM images of chitosan samples are reported in Figure S5.

### 3.1. N_2_ and CO_2_ Adsorption Test

**Figure 10:**
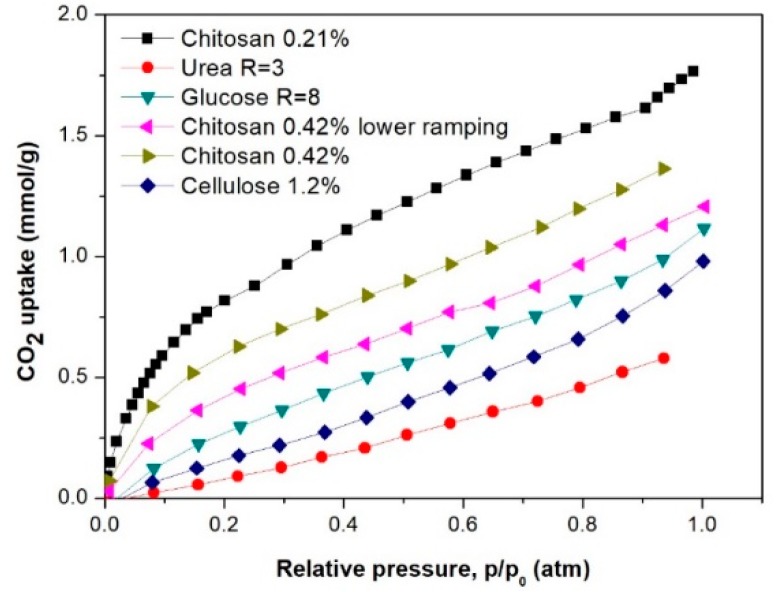
CO_2_ uptake vs. *p*/*p*_0_ for different samples.

N_2_ adsorption measurements were conducted to measure the surface area of the samples, while CO_2_ adsorption tests were performed to study the adsorption potential of our materials towards sensors applications. The adsorption capacity (*Ca*) was calculated using the formula reported below:

(1) $$Ca=\frac{\mathrm{Volume}\;\mathrm{of}\;\mathrm{gas}\;\left({\mathrm{CO}}_{2}\right)\;\mathrm{adsorbed}\;\left(\mathrm{cc}/g\right)}{\mathrm{Molar}\;\mathrm{volume}\;\left(\frac{\mathrm{cc}}{\mathrm{mmol}}\right)\;\mathrm{of}\;\mathrm{gas}\;\left({\mathrm{CO}}_{2}\right)}$$

where the volume of gas adsorbed corresponds to the *y*-axis and, for the adsorption capacity, the maximum value is considered, and molar volume is the volume occupied by one mole of a substance (chemical element or chemical compound) at a given temperature and pressure 1 atm. The obtained values are reported in millimoles of adsorbed gas per gram of the adsorbent. The molar volume of CO_2_ at standard conditions (273 K and 1 bar) is equal to 22.4 L/mol [23]. The results are reported in Table 2 and Figure 10.

Figure 10 shows that chitosan samples possess higher adsorption ability for CO_2_. From the comparison of chitosan-derived samples with different chitosan amount, we can safely say that the better adsorbance is not just a question of surface area, with chitosan 0.42% samples having a higher surface area than chitosan 0.21%.

By comparing the results achieved from CO_2_ adsorption with the results reported in the literature (see Table 3), it can be said that our materials nicely compete with those, with the advantage of being magnetic and, therefore, easily recoverable, but also having potential in magnetic sensor applications.

**Figure 11:**
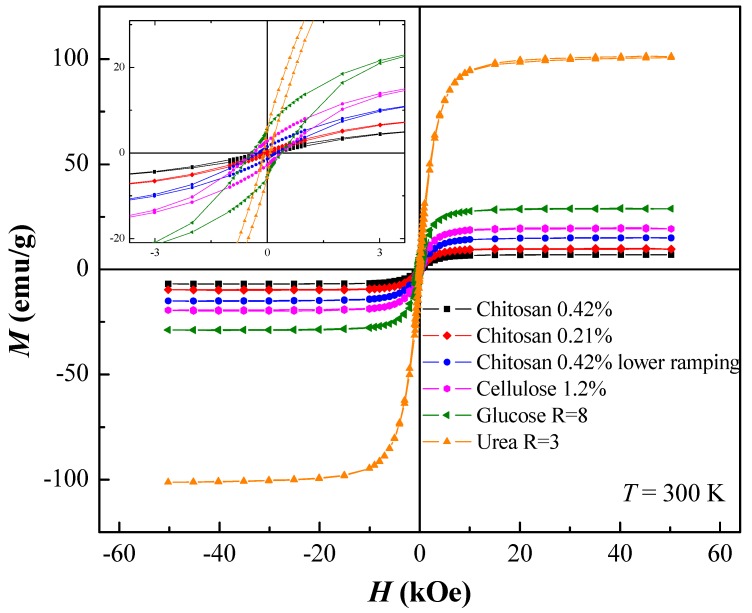
*M* vs. *H* curves collected at *T* = 300 K for the different samples.

To observe whether the carbon source has an influence on the magnetic behavior of the as-prepared samples, magnetometry measurements were performed at 300 K, and the results reported in Figure 11 and Table S1. Different magnetization behaviors are observed for samples prepared with varying carbon source, as well as metal/C-source ratio which, in turn, means that the different materials have the potential to be used in a range of different applications. The magnetization of all the samples studied was saturated in applied fields above ~10 kOe. The saturation magnetization, σM, varies from 6 emu/g for chitosan 0.42% up to 100 emu/g for urea *R* = 3 sample. All samples exhibit some magnetic hysteresis (see inset of Figure 11 and Table S1) with coercive fields ranging from 87 up to 400 Oe. 

It must be noted that magnetization and magnetic hysteresis are dependent on the amount of carbon present in the sample, and all samples reported here have a comparable magnetic material/carbon ratio by mass (the only exception is the urea sample, with the lowest carbon amount) as reported in Table 1.

### 3.2. Dyes and Oil Uptake

Fe_3_C-based nanocomposites were tested for possible applications as sorbent for contaminants in wastewater. Samples were treated with two different dyes as model molecules, i.e., methyl orange (MO) and methylene blue (MB). MO and MB were selected as model systems since they are well-known materials and can be easily quantified via UV-Vis spectroscopy. 

Furthermore, MB is a cationic dye while MO is an anionic dye. This ensures that the filtration is not solely based on electrostatic interaction. 

**Figure 12:**
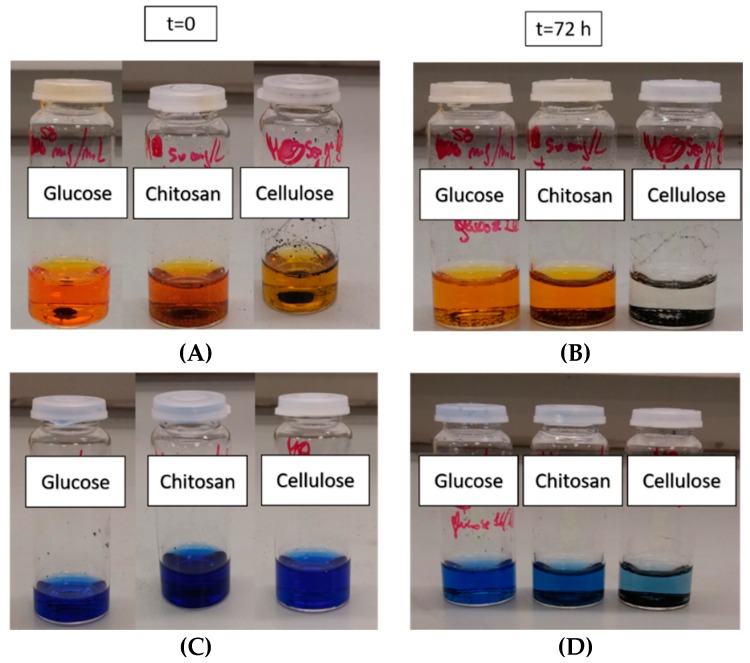
Dye sorption on Fe_3_C samples over time, with a concentration of 50 mg/L both for MO (**A**,**B**) and MB (**C**,**D**).

The solutions initially tested were 50 mg/L both for MO and MB and, after 72 h, a clear solution was obtained for cellulose sample in the case of MO, with a decrease in colour also for MB; such distinct differences were not observed for the other samples tested. The pictures are reported in Figure 12. Fe_3_C synthesized from cellulose was then believed to be the most promising candidate for dye removal from water.

Clearly, also in this case, surface area does not play the major role, and another factor should be responsible for the uptake.

As an example, the concentration over time calculated from UV–Vis analysis for MO is reported in Figure S6, and a decrease in the concentration of MO was observed for the first few hours, followed by the release of small quantity of dye over time.

To calculate the concentration of MO within the same conditions, a calibration curve was constructed, and the extinction coefficient (ε) was calculated. The calibration curve details can be found in Figure S7. The initial concentration of the solution without Fe_3_C is 7.64 × 10^−4^ M, so almost half of the MO was immediately absorbed in the material.

The recyclability of the compound was tested twice, and the sample was washed several times with water and ethanol to remove all the absorbed dye and dried. In all cases, a large amount of dye was released.

**Figure 13:**
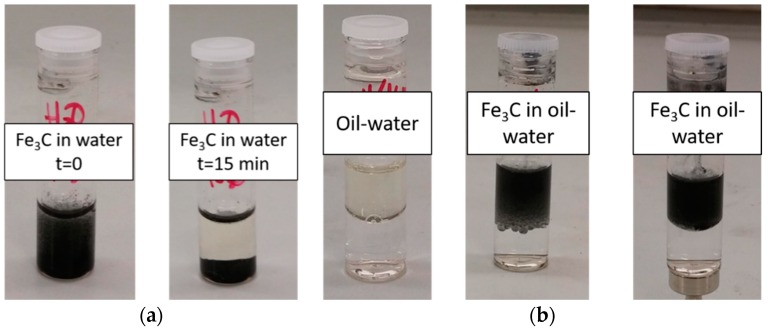
Pictures of the test of Fe_3_C sample from cellulose both in water (**a**) and in a mixture of water and vegetable oil (**b**). By using a magnet (see bottom of Fe_3_C in oil-water solution, picture on the far right), the contaminant is not carried into the water.

Vegetable oil was also tested as a real example of water contamination since it is commonly poured into the sink when used for cooking. Firstly, the sample was mixed with water and the powder almost immediately deposited on the bottom of the vial, with the addition of the oil, and the Fe_3_C sample (from cellulose) moved to the hydrophobic phase. When a magnet was moved close to the water phase, no oil mixed with it. The test with oil is reported in Figure 13. This material could then be applied for contaminants in wastewater, such as cooking oil, since it leaves the water ‘clean’.

## 4. Conclusions

Iron carbide@carbon nanocomposites were prepared using different carbon sources, with the aim of controlling morphology, surface area, and absorption properties of the final material. After characterization, the materials were tested for different applications, such as CO_2_ adsorption uptake, and dye and oil removal from water. The best CO_2_ adsorption performance was found for samples prepared from complex carbon source structures, such as chitosan, with a variation in the CO_2_ uptake by varying the quantity of chitosan used and the heat treatment; this finding is especially interesting because no direct relation with the surface area was observed. Further investigation over the CO_2_ adsorption behaviour of these samples was also studied to extend Fe_3_C-based materials for CO_2_ sensors, especially considering that, to the best of our knowledge, this was the first time where CO_2_ uptake was reported. Higher saturation magnetization was observed for urea *R* = 3 sample but, for the other nanocomposites, the presence of extra carbon should be taken into account.

The best sample for dye uptake was the one prepared from cellulose, with an almost immediate uptake of dye by the nanocomposite powder. This sample proved to be also applicable for oil removal from water with no powder present in the water. 

## Figures and Tables

**Table 1:** Experimental details of samples prepared using different carbon sources and heat-treated at 800 °C under nitrogen flow. *R* refers to the molar ratio between the carbon source and the metal-salt.

Carbon Source	Phases Present *	Average Crystallite Size (nm)		Elemental Analysis (wt %) ^#^
		by PXRD **	by TEM	
Urea *R* = 3	Fe_3_C (76%) (Fe^0^ (24%))	*N*/*A*	10–30	*N*% = 0.15, *C*% = 41.3
Cellulose 1.2%	Fe_3_C	36	30–80	*N*% = 0, *C*% = 87
Glucose *R* = 8	Fe_3_C (76%) (Fe^0^ (24%))	1724 (Fe^0^)	20–60	*N*% = 1, *C*% = 97.4
Glucose *R* = 16	Fe_3_C	63	15–50	*N*% = 0, *C*% = 90
Sucrose *R* = 8	Fe_3_C (78%) (Fe^0^ (22%))	36	10–40	*N*/*M*
Sucrose *R* = 10	Fe_3_C (72 (Fe^0^ (28%))	81	10–45	*N*/*M*
Sucrose *R* = 12	Fe_3_C (71%) (Fe^0^ (29%))	8655 (Fe^0^)	10–50	*N*/*M*
Chitosan 0.42%	Fe_3_C	73	10–25	*N*% = 2.3, *C*% = 82.7
Chitosan 0.21%	Fe_3_C	60	6–30	*N*/*M*
Chitosan 0.42% slower heating	Fe_3_C	46	10–40	*N*% = 6, *C*% = 94

*: For the samples including Fe^0^, the percentages of the two phases, calculated using the software Match!^®^, are reported between brackets. **: Calculated on the main peak of the Fe_3_C PXRD pattern using the Scherrer equation. For Fe_3_C phase, calculation refers to the peak with *d* value of 2.06682 Å that corresponds to plane (102). For Fe^0^ phase, calculation refers to the peak with *d* value of 2.02671 Å that corresponds to plane (110). ^#^:Theoretical value *C* wt %: 22%. *N*/*M* not measured.

**Table 2:** Surface area and CO_2_ adsorption (CO_2_ ads) values for the samples prepared.

Carbon Source	Surface Area (m^2^/g)	Max CO_2_ ads (mmol/g)
Urea *R* = 3	43	0.58
Cellulose 1.2%	211	0.98
Glucose *R* = 8	239	1.11
Chitosan 0.42%	131	1.37
Chitosan 0.21%	55	1.76
Chitosan 0.42% slower heat ramping	194	1.2

**Table 3:** Reported values of CO_2_ adsorbed (ads/mmol/g) for different materials.

Material	Max CO_2_ ads (mmol/g) (Temperature of Analysis)	Surface Area (m^2^/g)	Reference
AMC *	4.66 (273 K)	2475	[24]
CeO_2_–AMC	3.13 (273 K)	1906	
CuO–AMC	2.40 (273 K)	2097	
NiO–AMC	3.30 (273 K)	2006	
Mesoporous MgO	1.82 (not reported)	Not reported	[25]
Non-porous MgO	0.45 (not reported)	Not reported	
AC **	2.61 (298 K)	Not reported	[26]
CNTs	1.08 (323 K)	Not reported	
MgAlO	1.13 (623 K)	161	[27]
CaAlO	0.87 (623 K)	26	
N–rGO–ZnO	3.55 (298 K)	1122	[28]
MOF–Mg ***	1.1 (298 K)	Not reported	[29]
MOF–Cu	0.48 (298 K)	Not reported	
MOF–Fe	1.1 (298 K)	Not reported	
ZnO–nanoporous carbon	4.42 (273 K)	706	[30]
MCM-41	0.62 (298 K)	1129	[31]
Meso Al_2_O_3_	0.84 (298 K)	271	

*: AMC = activated mesoporous carbon. **: AC = activated carbon. ***: MOF = metal organic frameworks.

## Supplementary Materials

The following are available online at http://www.mdpi.com/1996-1944/12/2/323/s1, Figure S1: Particles dimension histograms for the samples discussed in the manuscript. (A) urea *R* = 3, (B) cellulose, (C) glucose *R* = 8, (D) glucose *R* = 16, (E) sucrose *R* = 8, (F) sucrose *R* = 10, (G) sucrose *R* = 12, (H) chitosan 0.42%, (I) chitosan 0.21%, (L) 0.42% chitosan with slower rate, Figure S2: SEM images of samples prepared with (A) urea and (B) cellulose respectively, Figure S3: SEM images of samples prepared using glucose with different glucose/iron molar ratio (A) *R* = 8 and (B) *R* = 16, Figure S4: SEM images of samples prepared using sucrose with different sugar/iron molar ratio: (A) *R* = 8; (B) *R* = 10; (C) *R* = 12; (D) *R* = 16, Figure S5: SEM images of samples prepared with different amount of chitosan (A) 0.42%, (B) 0.21%, (C) 0.42% slower heat ramping, Figure S6: Variation in the concentration of MO in cellulose sample over time, Figure S7: Calibration curve for methyl orange, in the insert details of the linear regression are reported, Table S1: Coercive field, *H*_c_, and the saturation magnetization, σ_M_, per gram at *T* = 300 K for the different samples studied.
