# Supplementary material for: Iron Carbide@Carbon Nanocomposites: A Tool Box of Functional Materials
Source: Materials (Basel). 2019 Jan 21;12(2):323. doi: 10.3390/ma12020323 (PMC6356575; doi:10.3390/ma12020323)
Supplement: Supplementary file 1 [file materials-12-00323-s001.pdf]

materials

MDPI

Supplementary Materials

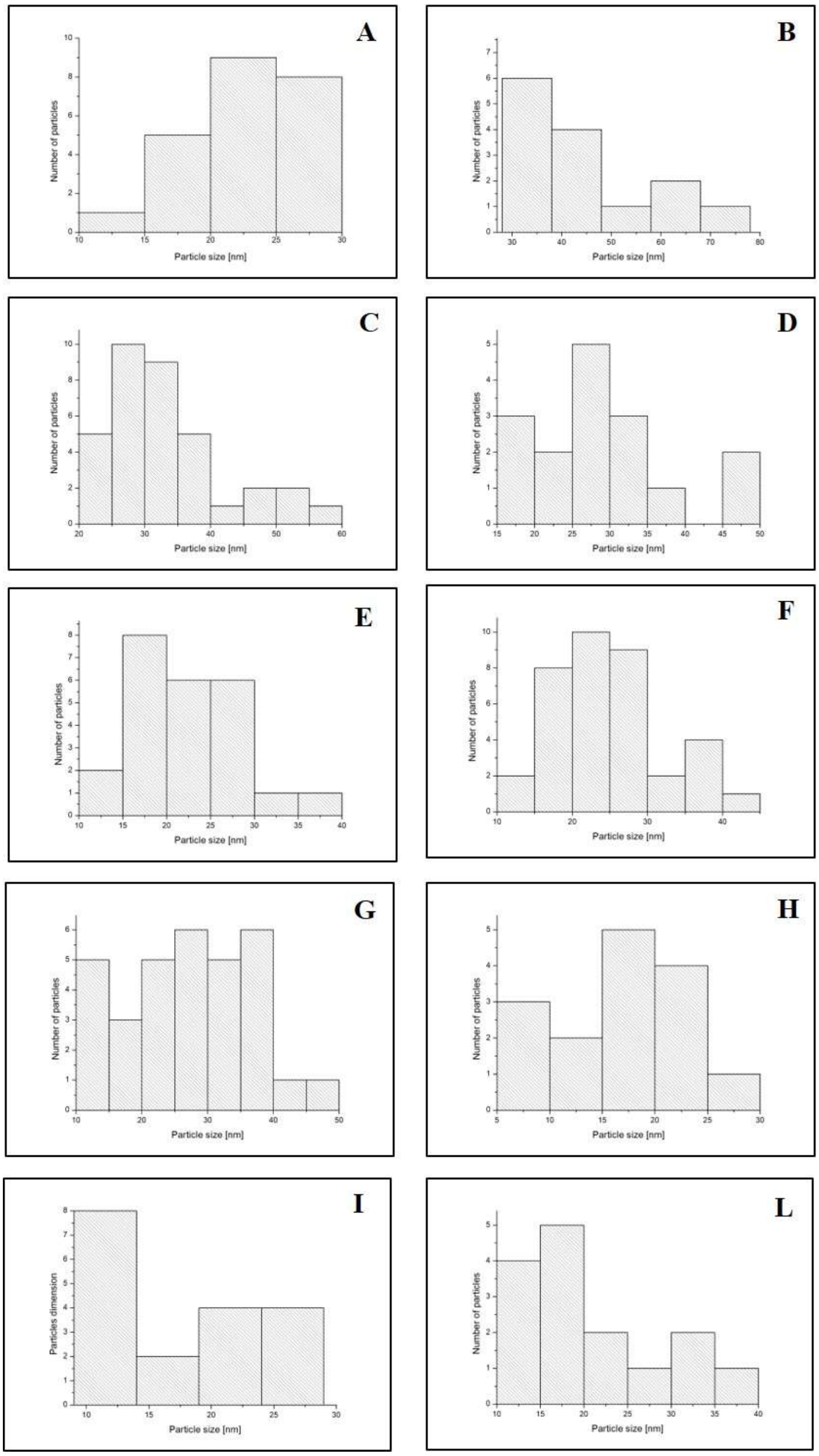

Figure S1. Particles dimension histograms for the samples discussed in the manuscript. (A) urea *R* = 3, (B) cellulose, (C) glucose *R* = 8, (D) glucose *R* = 16, (E) sucrose *R* = 8, (F) sucrose *R* = 10, (G) sucrose *R* = 12, (H) chitosan 0.42%, (I), chitosan 0.21%, (L), 0.42% chitosan with slower rate.

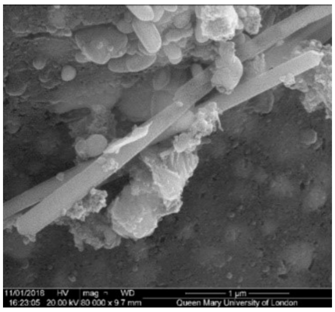

(**A**) (**B**)

Figure S2. SEM images of samples prepared with (**A**) urea and (**B**) cellulose respectively.

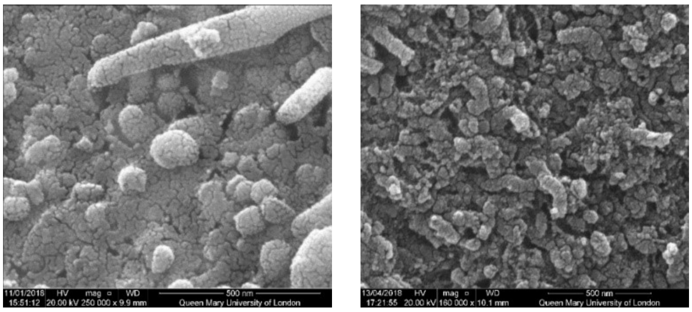

(**A**) (**B**)

Figure S3. SEM images of samples prepared using glucose with different glucose/iron molar ratio (**A**) $R = 8$ and (**B**) $R = 16$.

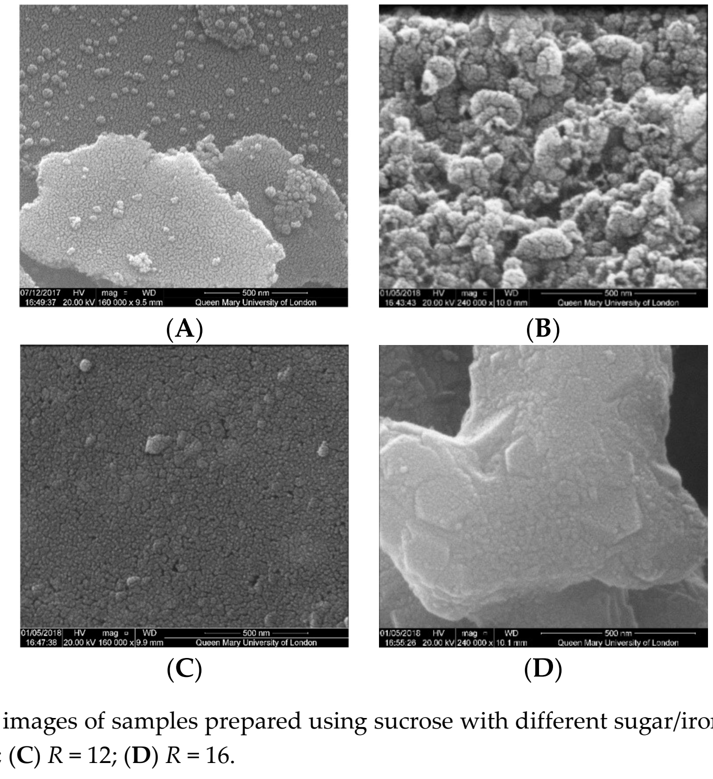

(**A**) (**B**)

(**C**) (**D**)

Figure S4. SEM images of samples prepared using sucrose with different sugar/iron molar ratio: (**A**) $R = 8$; (**B**) $R = 10$; (**C**) $R = 12$; (**D**) $R = 16$.

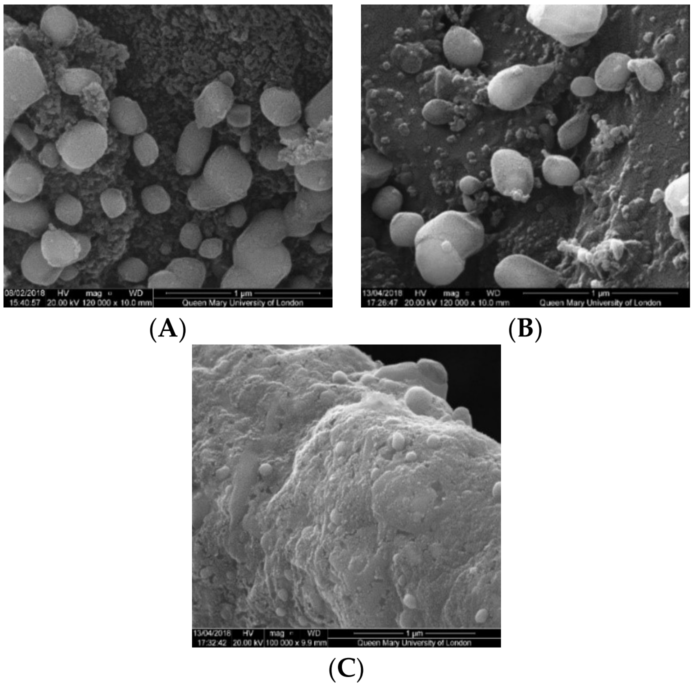

Figure S5. SEM images of samples prepared with different amount of chitosan (**A**) 0.42%, (**B**) 0.21%, (**C**) 0.42% slower heat ramping.

Table S1. Coercive field, H_c, and the saturation magnetization, σ_M, per gram at T = 300 K for the different samples studied.

Sample name	Coercive field H_c (Oe)	σ_M (emu/g)
Urea $R = 3$	87 (3)	100
Cellulose 1.2%	375 (5)	20
Glucose $R = 8$	400 (10)	28
Chitosan 0.21%	90 (3)	10
Chitosan 0.42%	200 (5)	6
Chitosan 0.42% slower heat ramping	240 (5)	15

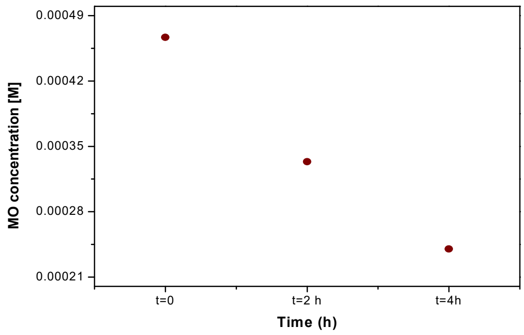

Figure S6. Variation in the concentration of MO in cellulose sample over time.

Calibration curve for methyl orange (MO) was done as follow by dilution. Starting from a stock solution of 250 mg/L, four solutions of different known concentrations of 0.04, 0.12, 0.2 and 0.27 mmol, were prepared. Their absorbance at λ = 461 nm was recorded with UV-Vis spectroscopy.

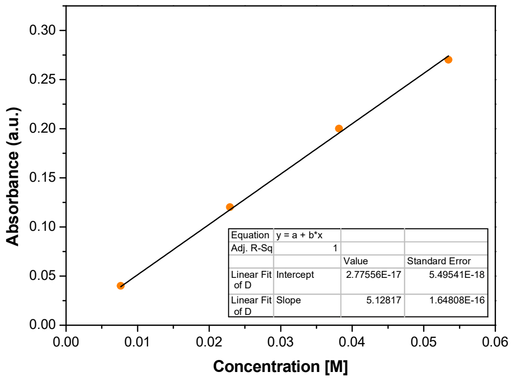

Figure S7. Calibration curve for methyl orange, in the insert details of the linear regression are reported.

Considering a cuvette with path length of 2 mm, the extinction coefficient was calculated to be $\varepsilon = 2.5 \cdot\times 10^4$.
